# Will They Stay or Will They Go? Motivational Profiles, Retirement-Related Correlates, and Retirement Intentions Among 58–72-Year-Old Workers

**DOI:** 10.3389/fpsyg.2022.807752

**Published:** 2022-03-03

**Authors:** Hallgeir Halvari, Anja H. Olafsen

**Affiliations:** Department of Business, Marketing and Law, University of South-Eastern Norway, Kongsberg, Norway

**Keywords:** self-determination theory, motivation profiles, autonomous work motivation, controlled work motivation, amotivation, retirement

## Abstract

Demographic changes indicate that the number of people aged 60 years and above will double in the next 30 years, and politicians around the world have an interest in delaying retirement in order to benefit national economies by lowering the burden on public pension systems. In this study, it is examined whether and how combinations of multiple types of work motivation based on self-determination theory (SDT) would be associated with retirement-related factors and retirement intentions. Using a person-centered approach to identify latent work motivation profiles among older workers, four profiles emerged: (1) the Low Motivation Profile with below-average levels on most motivational regulations, but in particular, lack identified work regulation; (2) the Autonomous Motivation Profile with higher levels of autonomous motivation and lower levels of controlled motivation and amotivation; (3) the High Motivation Profile with simultaneously high autonomous and controlled motivation; (4) the Amotivated Profile. Compared to the Low Motivation and Amotivated Profiles, the Autonomous and the High Motivation profiles show higher levels of vigor and lower levels of job stress, exhaustion and turnover-, and retirement intentions. However, the High Motivation Profile seems to pay a cost because they experience significantly more job stress than employees in the Autonomous profile. In addition, variable-based correlations showed higher levels of vigor and lower levels of job stress, exhaustion, and turnover intentions to be associated with lower levels of retirement intentions. The results are discussed in relation to managers and organizational endeavors to rebuild lost work identification and reduce extrinsic work motivation and amotivation in order to motivate older workers to stay longer at work.

## Introduction

United Nations (2015) prospects indicate that the number of people aged 60 years and above will double in the next 30 years. Population aging is associated with various challenges at the social, psychological, cultural, political, and economic levels. For instance, politicians have an interest in delaying retirement in order to benefit national economies by lowering the burden on public pension systems. At the same time, the industrial workforce and economy would be strengthened by keeping the older employees’ skills and experiences longer (Henning et al., 2019; Galkute and Herrera, 2020). In Norway, the government released the Norwegian Pension Reform in 2011 (Fredriksen et al., 2019), and subsequently, in 2015, the mandatory retirement age was increased from 70 to 72. It was believed that this would be instrumental in retaining more people at work for longer, and hence, benefit the economy. Concerns related to demographic changes associated with economic and workforce challenges are similar in countries around the world (Van den Berg, 2011; Brusch and Büsch, 2013; United Nations, 2015; Henning et al., 2019).

Thus, the question becomes how to best stimulate more people to stay longer at work? The causes of early retirement or extended work are complex. Factors in the working environment interact with factors related to individual health and personal economy, family, close relationships, leisure needs, planning and preparation, motivation to retire or continue working, and social security systems available (Shultz et al., 1998; Noone et al., 2013; Midtsundstad et al., 2017; Fouquereau et al., 2018).

Interviews of managers indicate that frequent reorganizations accompanied by restructuring at work, new demands for employees, and increased pressure and stress are related to exhaustion and subsequent work exit (Herlofsen and Hellevik, 2019). However, in the same article, the quantitative data of a large sample revealed that older workers did not perceive themselves as more exhausted than younger workers. The figures were actually the other way around. In addition, the correlation between exhaustion and work-exit among 60–67-year-olds was not significant. Other research supports these findings, as exhaustion was not a central reason for early work exit in the public sector. Loss of motivation and the need for more leisure time were more important (Midtsundstad, 2005). Because exhaustion, job stress, and turnover intentions are closely positively associated (Chung et al., 2017; Kim, 2018) and linked to low intrinsic motivation (Kim, 2018), motivation might be misperceived with these constructs, in particular, among older workers. This reasoning is supported in a Dutch study (Henkens and Leenders, 2010) linking motivational constructs such as lack of work challenges, lack of autonomy, and lack of support from colleagues to exhaustion, which, in turn, predicted plans of early work exit. In sum, this research indicates that work motivation may play a central role in employees’ desire to stay at work.

Yet to date, no study has examined the combination of different work motivation regulations among older workers, and how they are associated with factors like job stress, emotional exhaustion, and turnover intentions supposed to affect retirement intentions. Thus, aligned with a motivational framework embedded in self-determination theory (SDT) (Deci and Ryan, 2000; Ryan and Deci, 2017), the purpose of this study is twofold: (1) to determine if it is possible to identify unique latent profiles based on older employees work motivation regulations, and (2) determine if latent motivation profiles are associated with employees’ vigor, emotional exhaustion, job stress, turnover intentions, and retirement intentions. Acknowledging the multitude of factors involved in senior employees’ decisions to retire or continue working, the present study will focus on contemporary conceptualizations and measures of work motivation, controlling for personal and household income, and financial security.

### Work Motivation

To the extent that employees autonomously engage in their work environment, SDT proposes that they will tend to internalize and integrate the behavior, values, beliefs, attitudes, and learning contents they encounter within that environment (Williams and Deci, 1996; Deci et al., 2017). SDT posits that the regulation of behavior varies to the extent it is autonomous vs. controlled. Autonomously motivated behavior is characterized by employees who experience a sense of psychological freedom and choice in carrying out an activity; their behavior is often self-initiated and consistent with their personal values and interests, such that they feel as though the behavior emanates from themselves (de Charms, 1968). When employees are autonomously motivated, it is suggested that they are more self-regulated toward willingly adopting values and beliefs proposed at work and in their profession.

According to SDT, two types of autonomous work motivation are differentiated, that is, intrinsic work motivation and well-internalized extrinsic identified work motivation. *Intrinsic* work motivation represents the prototype of autonomous motivation and is defined as doing an activity for its own sake, namely, because it is interesting, exciting, and enjoyable in itself. Some employees may not be intrinsically motivated for work. However, they might still do so willingly, given that they believe their work is personally meaningful and valuable to them. This represents the second type of autonomous motivation, labeled *identified* work motivation. Conversely, controlled work motivation is less self-determined or internalized and involves doing an activity because the employee feels pressured or forced to do so by an external or internal force, such as receiving rewards and approval or avoiding punishments or criticism. SDT specifies two subtypes of controlled work motivation, that is, *introjected* work motivation, which is prevailing when employees comply with some partially internalized expectations that are buttressed with threats of guilt, shame, anxiety, or self-esteem contingencies. The least self-determined work motivation is labeled *external*, that is when complying with the demands of others. For instance, some employees feel pressured to engage in their work because they have financial problems or because they are afraid to lose their job or feel guilty if they do not comply with the leader’s expectations. Whether the demands are wholly external or have been partially internalized such as with introjection, in both cases, individuals feel that they have no choice but to engage in the activity (Deci and Ryan, 1985, 2000). Finally, *amotivation* is described as the complete absence of self-determination and intention to behave, in which the employee perceives that no behavior would reliably lead to desired outcomes or because they believe they could not successfully effectuate a behavior that would lead to the outcome (Deci and Ryan, 2000). Hence, we now turn to a closer look at how these motivational regulations have been associated with relevant work-related outcomes.

### Work Motivation, Retirement Antecedents, and Retirement Intentions

In the present study, vigor (being the antipode of emotional exhaustion; Schaufeli et al., 2006), emotional exhaustion, job stress, and turnover intentions are labeled close retirement antecedents. This is because the literature has linked sustained work or late retirement to low job stress (Nielsen Breidahl, 2011), low emotional exhaustion (Nielsen Breidahl, 2011; De Wind et al., 2017; Stynen et al., 2017), and low turnover intentions (Hofstetter and Cohen, 2014). In addition, higher levels of emotional exhaustion, job stress, and turnover intentions are positively correlated (Olafsen et al., 2016; Chung et al., 2017; Kim, 2018) and they are all associated with low autonomous or intrinsic motivation or high need frustration (Williams et al., 2014; Olafsen et al., 2016, 2021; Chung et al., 2017; Kim, 2018). Thus, theoretically, work motivation may be a more distal construct affecting both the close antecedents of retirement and the retirement intentions themselves.

Research grounded in SDT related to sustained work is very limited in the literature. The research conducted is unidimensional and have operationalized work motivation with question wordings of job tasks or work being “important,” “interesting,” and/or “rewarding” (Midtsundstad and Nielsen, 2014; Ang et al., 2016; Nilsson, 2017), or used a measure of intrinsic motivation (Van den Berg, 2011), indicating that autonomous motivation is important for sustained work. Others have operationalized motivation in a universal way with one item asking older workers to rate the statement “My work motivation is very high” (Büsch et al., 2010; Brusch and Büsch, 2013), and consequently, we do not know whether the motivation measured is autonomous, controlled, or amotivated. What is known is that total motivation has been related to older workers’ intentions to continue working after reaching the retirement age in Germany (Büsch et al., 2010; Brusch and Büsch, 2013). Accordingly, a systematic literature review (Galkute and Herrera, 2020), using the two German studies referred to above, concluded that intrinsic motivation is strongly positively related to post-retirement work. However, this is a problematic conclusion because these studies did not measure intrinsic motivation. Conversely, multidimensional motivation theory, as operationalized from SDT (Ryan and Deci, 2017), has been used in one study retrieved, indicating that retirees who are intrinsically motivated for their work seem to benefit from continuing working in retirement due to increases in relatedness satisfaction (Henning et al., 2019). In sum, with only one study retrieved using SDT and multidimensional measures of motivation, the knowledge gap on the relations between work motivation and continued work after retirement age is large.

### Work Motivation Profiles and Work-Related Outcomes

Given the multidimensional view of motivation afforded by SDT, a current trend in the literature is using person-centered studies to examine how patterns of motivational regulations relate to predictors and outcomes of interest (Olafsen and Deci, 2020). Because people will have varying levels of the different regulations, understanding how the various combinations of these regulations can contribute to explaining work exit and associated correlates among older workers, would be valuable. However, to our knowledge, person-centered studies using work motivation profiles in relation to continued work after retirement age are currently absent in the literature. Consequently, we draw on existing studies using latent profiles of work motivation to inform us about: (1) what profiles we might expect to find, and (2) how these profiles might be associated with work-related outcomes such as vigor, emotional exhaustion, job stress or strain, and turnover intentions in the general working population. These work-related outcomes might indicate close antecedents of continued work after retirement age. Similar to the present study, two previous person-centered studies have used the full version of the Multidimensional Work Motivation Scale (MWMS) (Howard et al., 2016; Fernet et al., 2020). Hence, these studies are considered first, followed by a review of studies that have omitted parts of the MWMS or used older versions of the scale.

In the first study, among employees from the technology, government, and manufacturing sectors, Howard et al. (2016) reported: (a) an autonomous motivated or self-determined profile (viz., higher levels of autonomous work motivation, lower levels of controlled work motivation, lower levels of amotivation); (b) an autonomous and controlled motivation profile (viz., both higher levels of autonomous and controlled types of regulations, and lower amotivation); (c) a balanced or poorly motivated profile (viz.: low to average scores on all motivational regulations); (d) an amotivated profile (viz., higher amotivation and low to average levels of all other motivational regulations). In this study, workers in the autonomous motivated profile and those in the autonomous and controlled motivated profile yielded superior work-related outcomes (viz., lower levels of burnout and emotional exhaustion, and higher levels of engagement, job satisfaction, extra-role performance, in-role performance) (Howard et al., 2016).

In the second study among nurses, latent profile and latent transition analyses revealed four distinct profiles based on global self-determination (viz., autonomy) and the specific behavioral regulations. Two profiles: (1) one with very high global self-determination combined with high autonomous motivation (viz., identified regulation), average intrinsic motivation, and high controlled motivational regulations, and (2) the profile with a moderately high level of global self-determination combined with average levels of identified, introjected and external regulations, and amotivation, but low levels of intrinsic motivation. These two profiles were associated with lower emotional exhaustion and turnover intentions and with higher in-role performance compared to more poorly motivated profiles (Fernet et al., 2020).

Other studies using the MWMS also yielded four motivational profiles, indicating that the profile with the highest level of autonomous motivation and lower levels of controlled motivation displayed the most desired work-related outcomes compared with the less autonomous and more controlled motivation profiles among soldiers (Gillet et al., 2017), among employees from various companies (Gillet et al., 2018), and among managers (Graves et al., 2015). Four profiles were also found in other studies, in which profiles of both high autonomous motivation/low controlled motivation, and high autonomous motivation/high controlled motivation, have been associated with more desired work-related outcomes when compared with profiles characterized with lower autonomous motivation among employees in a consulting company, a community organization (Van den Broeck et al., 2013), and among teachers (Van den Berghe et al., 2014).

### Hypotheses

Although none of the person-centered studies described above focus directly on retirement intentions or willingness to continue working after the retirement age, they include vigor or job engagement, emotional exhaustion, burnout, perceived strain or job stress, and turnover intentions which are supposed to be close antecedents associated with retirement intentions.

Accordingly, in the current study, we used a person-centered approach to identify latent profiles based on older workers’ motivation regulations. Then, we used membership in the latent profiles to study their levels of vigor, emotional exhaustion, job stress, turnover intentions, and retirement intentions. Based on the literature reviewed, we hypothesized that profiles with higher levels of autonomous motivation (viz., higher levels of intrinsic motivation and identified regulations) combined with lower or higher levels of controlled motivation (viz., introjected and external regulations), and lower levels of amotivation will report more vigor, less exhaustion, less job stress, less turnover intentions, and less retirement intentions than less autonomous latent profiles. In addition, we hypothesized that retirement intentions would be negatively associated with vigor and positively associated with exhaustion, job stress, and turnover intentions.

## Materials and Methods

### Participants

The participants were 500 older workers aged from 58 to 72 years in a representative online panel for Norway regarding gender, age, geography, and type of work. The participants completed the survey in the fall of 2020. Informed consent was received from each participant before they completed the survey. The participants’ mean age was 62.43 years (*SD* = 3.32), with an equal distribution of males (50.2%) and females, and 70.2% of them were married or lived together with a close friend/cohabitant. Fulltime work was performed by 72.4% of the participants, whereas the rest worked part-time above (17.2%) or below (10.4%) a 50% position. Of the participants, 60.6% reported that they worked a normal daytime, 26% worked a combination of day and evening time, while 13.4% had shift and night work. Their average tenure was 40.24 years (*SD* = 6.01), and their average personal income/year is estimated to be about 600,000 NOK or about 71,000 USD.

### Measures

All non-Norwegian measures were translated into Norwegian and then back-translated into English using the approach recommended by Beaton et al. (2000).

#### Work Motivation

The MWMS (Gagné et al., 2015) was used to assess seven distinct motivational regulations. Each item is an answer to the question “Why do you or would you put effort into your current job?” along with a 1 (not at all) to 7 (completely) point Likert scale. Sample items include, “I don’t know why I’m doing this job, it’s pointless to work” (Amotivation; Cronbach’s α = 0.63 for the three items), “Because others will reward me financially only if I put enough effort in my job (e.g., employer, supervisor…)” (External regulation material; α = 0.85 for the three items), “To get others’ approval (e.g., supervisor, colleagues, family, clients…)” (External regulation social; α = 0.85 for the three items), “Because I have to prove to myself that I can” (Introjection regulation approach; α = 0.77 for the two items), “Because otherwise, I will feel ashamed of myself” (Introjected regulation avoidance; α = 0.89 for the two items), “Because putting efforts in this job aligns with my personal values” (Identified regulation; α = 0.89 for the three items), and “Because the work I do is interesting” (Intrinsic motivation; α = 0.95 for the three items). The MWMS has been validated in seven languages and nine countries, including Norwegian (Gagné et al., 2015).

#### Vigor

Vigor was assessed with the short version of the Utrecht Work Engagement Scale (Schaufeli et al., 2006). Responses to the nine items (e.g., “At my work, I feel bursting with energy”) were made on a scale ranging from 1 (never) to 7 (daily). The reliability for this measure was α = 0.94.

#### Emotional Exhaustion

Emotional exhaustion was assessed with the five-item emotional exhaustion subscale of the Maslach Burnout Inventory (Maslach et al., 1996). A sample item is: “I feel burned out from my work.” Responses were made on a 7-point scale from 1 (never) to 7 (always). The reliability for this measure was α = 0.88.

#### Job Stress

Job stress was assessed with ten items from the Perceived Stress Scale (Cohen et al. (1983)) adapted to the job context. Sample item: “In the last month, how often have you been upset because of something that happened unexpectedly at work?” Responses were made on a 5-point scale ranging from 0 (never) to 4 (very often). The reliability of this scale was α = 0.81.

#### Turnover Intentions

Turnover intentions were assessed by three items focusing on the participants’ current thinking about turnover (O’Driscoll and Beehr, 1994); sample item: “I am thinking of leaving this job.” Responses were made on a 7-point scale from 1 (strongly disagree) to 7 (strongly agree). The reliability for this measure was α = 0.78.

#### Retirement Intentions

Retirement intentions were measured by three questions regarding retirement planning. The items are: “I am thinking of retiring from this job”; “I plan to retire during the next 12 months”; “I work actively to retire.” Responses were made on a 7-point scale from 1 (strongly disagree) to 7 (strongly agree). The reliability for this measure was α = 0.86.

#### The Control Variables

The control variables were gender (male; female), age (years), personal and household income (NOK), financial security, and planned length of working life (years). A brief measure of perceived financial security was used (Munyon et al., 2020). The items were: “I have adequate income”; “I have adequate credit”; “I have financial stability”; “I have enough savings for an emergency”; “I have enough assets.” A 7-point scale was used ranging from 1 (strongly disagree) to 7 (strongly agree). The reliability for this measure was α = 0.84. For “planned length of working life,” the following question was applied: “How long do you plan to be working?” The responses ranged from “less than a year” to “10 years more.”

### Data Analysis

Latent profile analysis (LPA) was conducted in Mplus version 8.6 (Muthén and Muthén, 1998-2020) to explore and identify different subgroups within the sample based on the participants’ responses to the motivational regulations (Berlin et al., 2014). The models were specified using the maximum likelihood estimator and a stepwise comparison of models was conducted when evaluating a one-profile model with successively more profiles (Nylund et al., 2007).

Models with one to six profiles were analyzed and evaluated based on a combination of goodness of fit (GOF) indices and profile sizes (> 5%) (Jung and Wickrama, 2008). First, the Akaike’s information criterion (AIC) (Akaike, 1987), Bayesian information criteria (BIC) (Henson et al., 2007), and sample-size adjusted BIC were inspected and compared across the solutions to assess the model fit. For each of the indices, a lower value indicates a better fit to the data (Shibata, 1976; Yang, 2006; Henson et al., 2007). Second, the highest possible entropy was assessed to evaluate the classification accuracy. A value close to 1 indicates high accuracy in classification (Berlin et al., 2014). Third, the average latent class posterior probability was evaluated to assess class separation. Some researchers use a 0.80 cutoff for acceptable diagonal probabilities (Weden and Zabin, 2005). Others suggest a cutoff value of 0.90 or greater (Muthén and Muthén, 2000). While 0.90 is ideal, if the other criteria are met and the model is theoretically supported, probabilities between 0.80 and 0.90 are deemed acceptable (Weller et al., 2020). Fourth, a significant *p*-value on the bootstrap likelihood ratio test (BLRT) (McLachlan and Peel, 2000) and the Lo–Mendell–Rubin adjusted likelihood ratio test (LMR) (Lo et al., 2001) were used to determine whether the current solution had a statistically better fit (*p* < 0.05) to the data than the previous solution. Finally, both theoretical justifications and an evaluation of the substantial meaning of profiles were conducted by the researchers when deciding on the final number of profiles (Jung and Wickrama, 2008).

To test our hypothesis that profiles with higher levels of autonomous motivation will report more vigor, less exhaustion, less job stress, less turnover intentions, and less retirement intentions than less autonomous latent profiles, the automatic three-step Bolck, Croon, and Hagenaars (BCH) approach was used (Asparouhov and Muthén, 2014). This procedure offers an omnibus test that includes differences between profiles on each distal outcome (Bolck et al., 2004) yielding the least biased estimates in relation to comparative analysis (Bakk and Vermunt, 2016). In addition to determining the significant differences in the associated variables between the profiles, the effect size (ES) of these differences was explored and interpreted: Cohen’s *d* ES: 0.01–0.19 (very small), 0.20–0.49 (small), 0.50–0.79 (moderate), 0.80–1.19 (large), 1.20–1.99 (very large), and 2.00 and higher (huge) (Sawilowsky, 2009).

## Results

### Descriptive Statistics, Correlations, and Reliability

Table 1 presents the means, SDs, skewness values, and intercorrelations for the study variables. Relatively high levels of intrinsic motivation, identified regulation, and vigor, and low levels of amotivation, exhaustion, and turnover intentions, were reported. The reliability coefficients for the measures were acceptable (Nunnally, 1979). Table 2 contains the descriptive statistics and Pearson correlations for the retirement intentions and correlates. The correlations between retirement intentions and personal income (*r* = 0.01), household income (*r* = −0.02), and financial security (*r* = −0.05) are all non-significant, indicating that the economy is of minor importance for retirement intentions among Norwegian senior employees. The same is the case for the alternative measure of retirement “planned length of working life.” This alternative measure of retirement was strongly correlated (*r* = −0.58) with the “retirement intention” measure used in the LPA, indicating that the measure used is valid.

**TABLE 1:** Descriptive statistics, Pearson correlations, and reliability coefficients in the diagonal (*N* = 500).

Variables	1.	2.	3.	4.	5.	6.	7.	8.	9.	10.	11.	12.	13.	14.
1. Intrinsic motivation	0.95													
2. Identified regulation	0.64***	0.89												
3. Introjection approach	0.21***	0.37***	0.77											
4. Introjection avoidance	0.05	0.30***	0.48***	0.89										
5. External social	–0.05	0.03	0.44***	0.35***	0.85									
6. External material	–0.05	–0.03	0.38***	0.25***	0.47***	0.85								
7. Amotivation	−0.23***	−0.26***	–0.07	0.07	0.12**	0.21***	0.63							
8. Vigor	0.61***	0.34***	0.11*	–0.05	–0.07	–0.05	−0.24***	0.94						
9. Job stress	−0.34***	−0.23***	0.09*	0.12**	0.16***	0.09*	0.27***	−0.39***	0.81					
10. Exhaustion	−0.30***	−0.09*	0.01	0.10*	0.05	0.01	0.10*	−0.39***	0.48***	0.88				
11. Turnover intentions	−0.32***	−0.17***	0.05	0.07	0.05	0.15***	0.19***	−0.32***	0.30***	0.34***	0.78			
12. Retirement intentions	−0.21***	−0.16***	–0.03	0.05	0.02	0.04	0.18***	−0.28***	0.19***	0.23***	0.35***	0.86		
13. Age	0.04	–0.02	–0.07	−0.10*	–0.05	–0.04	0.10*	0.05	−0.09*	−0.17***	−0.13**	0.27***	–	
14. Gender^a^	0.05	0.12**	0.04	0.07	–0.01	−0.10**	−0.12**	0.07	0.09	0.08	–0.03	−0.17***	−0.20***	–
*Means*	5.12	5.30	3.89	3.24	2.83	2.41	1.44	5.56	2.16	2.75	2.19	3.27	62.43	1.50
*Standard deviations*	1.32	1.20	1.38	1.56	1.19	1.24	0.72	1.23	0.51	1.34	1.39	1.91	3.32	0.50
*Skewness*	–0.80	–1.10	–0.28	0.20	0.35	0.85	1.86	–1.05	0.15	0.92	1.45	0.50	0.68	0.01

*p < 0.05; **p < 0.01; ***p < 0.001.

*^a^For gender: a value of 1 represents males and a value of 2 represents females. Spearman’s point-bi-serial correlations are used for gender.*

**TABLE 2:** Descriptive statistics and Pearson correlations for retirement intentions and control variables (*N* = 500).

Variables	1.	2.	3.	4.	5.
1. Retirement intentions	–				
2. Personal gross income per year	0.01	–			
3. Household gross income per year	-0.02	0.62***	–		
4. Financial security	−0.05	0.33***	0.31***	–	
5. Planned length of working life	−0.58***	0.05	0.03	−0.03	–
*Means*	3.27	3.59^a^	4.63^b^	5.53	3.48^c^
*Standard deviations*	1.91	1.21	1.38	1.07	1.71
*Skewness*	0.50	0.31	−0.61	−0.83	0.51

***p < 0.001.

*^a^Mean estimated to about 660,000 NOK.*

*^b^Mean estimated to about 860,000 NOK.*

*^c^Mean estimated to 4.5 years more.*

### Hypothesis Testing: Latent Profile Analysis

Based on the results of the stepwise comparison of models with one to six profiles, a four-profile solution was favored. The AIC, BIC, and adjusted Bayesian information criteria (ABIC) continued to decrease until the six-profile solution, but the LRM only showed significant results up to the three-profile solution. Still, the BLRT was significant for the four-profile solution, and in combination with the other acceptable GOF indices in terms of acceptable posterior probabilities and entropy, the four-profile solution was favored based on an evaluation of the substantial meaning and theoretical justification for the number of profiles (Jung and Wickrama, 2008). Also, the four-profile solution is in line with profiles on motivational regulations in previous studies (e.g., Howard et al., 2016).

**FIGURE 1:**
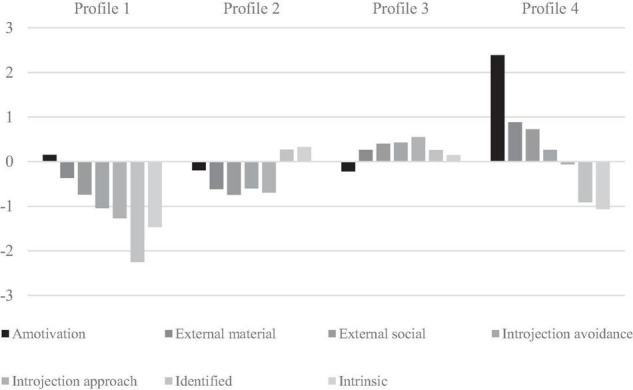
Standardized means for variables in profiles 1–4 based on the latent profile analysis.

As shown in Table 3, the proportions that reflected the most likely percentage of membership in Profiles 1–4 were 7.2, 30.0, 55.6, and 7.2%, respectively. The “Low motivation” profile 1 was characterized by small to modest below average levels on the various motivational regulations, except for identified regulation scores which were significantly much lower than in all other profiles (see CIs in Table 4 and profiles illustrated in Figure 1), yielding large to very large effect sizes from −1.16 (profile 1 vs. profile 4), −1.86 (profile 1 vs. profile 3), to −1.37 (profile 1 vs. profile 2). The “Autonomous motivation” profile 2 was characterized by high levels of autonomous motivation and low levels of controlled motivation and amotivation. The “High motivation” profile 3 was characterized by relatively high levels of all types of motivation with the exception of amotivation. The “Amotivated” profile 4 was characterized by small to modest levels on the various motivational regulations, except for amotivation scores which were significantly much higher than in all other profiles (see CIs in Table 4), yielding very large and huge effect sizes from 1.30 (profile 4 vs. profile 1), 2.01 (profile 4 vs. profile 2), to 2.32 (profile 4 vs. profile 3). In addition, the Amotivated profile was also characterized by significantly higher levels of external material regulation than all other profiles, yielding small to large effect sizes from 1.08 (profile 4 vs. profile 1), 1.06 (profile 4 vs. profile 2), to 0.24 (profile 4 vs. profile 3).

**TABLE 3:** Model fit indices for latent profiles based on motivational regulations.

Profiles	Log likelihood	#fp	Scaling	AIC	BIC	ABIC	Entropy	LMR	BLRT	Posterior probability	Latent profile proportions (%)
1	−5603.72	14	1.164	11235.44	11294.44	11250.00	–	–	–	1	500
2	−5437.97	22	1.252	10919.94	11012.66	10942.83	0.87	0.002	0.000	0.91/0.97	81/419
3	−5318.80	30	1.341	10697.59	10824.03	10728.81	0.83	0.004	0.000	0.90/0.93/0.92	118/332/50
4	−5217.66	38	1.394	10511.33	10671.48	10550.87	0.81	0.064	0.000	0.93/0.86/0.91/0.88	34/145/282/39
5	−5126.87	46	1.343	10345.73	10539.60	10393.60	0.90	0.117	0.000	0.90/0.98/0.93/0.95/0.98	79/102/267/17/35
6	−5063.12	54	1.456	10234.25	10461.84	10290.44	0.87	0.318	0.000	0.92/0.98/0.83/0.96/0.97/0.88	18/17/116/101/35/213

*Ran with separate dimensions for external and introjection. ABIC, adjusted Bayesian information criteria.*

**TABLE 4:** An overview of the four identified profiles.

	Profile 1 (6.8%)		Profile 2 (29.0%)		Profile 3 (56.4%)		Profile 4 (7.8%)	
Variable	M (SD)	CI 95%	M (SD)	CI 95%	M (SD)	CI 95%	M (SD)	CI 95%
Intrinsic motivation	3.18 (2.04)	[2.49, 3.87]	5.56 (2.53)	[5.14, 5.98]	5.32 (1.51)	[5.15, 4.48]	3.71 (1.75)	[3.16, 4.26]
Identification	2.60 (3.15)	[1.54, 3.66]	5.63 (1.93)	[5.31, 5.95]	5.62 (1.34)	[5.46, 5.77]	4.21 (1.25)	[3.81, 4.60]
Introjection approach	2.14 (2.16)	[1.42, 2.86]	2.93 (3.85)	[2.30, 2.56]	4.65 (2.18)	[4.40, 4.90]	3.81 (1.37)	[3.37, 4.24]
Introjection avoidance	1.62 (2.04)	[0.93, 2.32]	2.31 (2.41)	[1.91, 2.71]	3.91 (3.53)	[3.50, 4.31]	3.66 (1.31)	[3.24, 4.08]
External social	1.95 (1.81)	[1.33, 2.56]	1.94 (2.41)	[1.55, 2.34]	3.31 (2.18)	[3.05, 3.58]	3.70 (1.19)	[3.32, 4.07]
External material	1.95 (1.34)	[1.51, 2.39]	1.64 (1.81)	[1.36, 1.93]	2.74 (3.35)	[2.46, 3.01]	3.50 (1.50)	[3.03, 3.97]
Amotivation	1.55 (0.93)	[1.25, 1.86]	1.30 (0.72)	[1.18, 1.42]	1.29 (0.67)	[1.20, 1.37]	3.15 (1.44)	[2.71, 3.59]

The results from the BCH showed that retirement intentions were lowest in the Autonomous motivation profile and the High motivation profile, and highest in the Low motivation profile and the Amotivated profile (see Table 5). Furthermore, the levels of retirement intention differed significantly between the Low motivation profile and the Autonomous motivation profile, between the Autonomous motivation profile and the Amotivate profile, and between the High motivation profile and the Amotivated profile. Given that retirement intentions are not describing specific years of expected working life, we conducted an alternative BCH analysis including “planned length of working life in years.” This analysis favored a similar solution with the same interpretation as the results obtained with “retirement intentions” and gave us information about the differences in the years of planned working life between the profiles. The Autonomous and High motivation profiles planned to work for about 1.6–3.4 years longer (adjusted for standard errors) than the Low motivation profile and the Amotivated profile.

**TABLE 5:** Differences between profiles on distal outcomes.

Variable	Profile 1 M (SD)	Profile 2 M (SD)	Profile 3 M (SD)	Profile 4 M (SD)	1 vs. 2 X^2^/d	1 vs. 3 X^2^/d	1 vs. 4 X^2^/d	2 vs. 3 X^2^/d	2 vs. 4 X^2^/d	3 vs. 4 X^2^/d
Vigor	5.31 (1.71)	5.92 (1.47)	5.74 (1.28)	4.35 (1.86)	3.59*/0.38	1.97^ns^/0.28	5.08*/0.54	1.40^ns^/0.13	23.85***/0.94	19.90***/0.87
Job Stress	2.39 (0.45)	1.93 (0.60)	2.19 (0.54)	2.61 (0.59)	23.49***/0.87	5.24*/0.40	3.16*/0.42	16.76***/0.46	40.69***/1.14	16.89***/0.74
Exhaustion	3.14 (1.87)	2.52 (1.59)	2.73 (1.41)	3.47 (1.57)	3.07*/0.36	1.47^ns^/0.25	0.66^ns^/0.19	1.56^ns^/0.14	11.30**/0.60	7.56**/0.50
Turnover intentions	2.68 (1.70)	1.97 (1.58)	2.06 (1.43)	3.55 (1.77)	4.71*/1.04	4.08*/0.39	4.46*/0.50	0.29^ns^/0.06	25.55***/ 0.94	24.66***/0.93
Retirement intentions	3.93 (2.19)	3.01 (2.36)	3.16 (2.03)	4.51 (1.95)	4.49*/0.40	3.74*/0.36	1.35^ns^/0.28	0.35^ns^/0.07	16.43***/0.69	15.57***/0.68

*p < 0.05, **p < 0.01, ***p < 0.001, one-tailed tests. ^ns^ = non-significant.

For the remaining distal outcomes, the same pattern of levels was detected for the four profiles. That is, the negative outcomes (i.e., turnover intentions, exhaustion, and stress) were lowest in the Autonomous motivation profile, then more moderate in the High motivation profile and the Low motivation profile, and highest in the Amotivated profile. For the positive outcome (i.e., vigor) the result was in the reversed direction (i.e., highest in the Autonomous motivation profile and lowest in the Amotivated profile). For exhaustion, these levels differed significantly only between the Autonomous motivation profile and the Amotivated profile, between the High motivation profile and the Amotivated profile, and between the Autonomous motivation profile and the Low motivation profile. For turnover intentions, the levels differed significantly between most of the profiles. That is, the Low motivation profile was significantly higher in turnover intentions than the Autonomous motivation profile and the High motivation profile, and significantly lower than the Amotivated profile. While it was not detected, there is a significant difference in the levels of turnover intentions between the Autonomous motivation profile and the High motivation profile; both of these profiles were significantly lower in levels of turnover intentions than the Amotivated profile. Furthermore, the level of stress was significantly different when comparing all four profiles. The stress scores were lowest in the Autonomous motivation profile, subsequently followed with more moderate scores for the Low motivation profile and the High motivation profile, and with the highest scores in the Amotivated profile. Lastly, the level of vigor was significantly lower in the Amotivated profile compared to the other three profiles, and significantly lower in the Low motivation profile compared to the Autonomous profile, but the results did not show any significant differences between the Low motivation and High motivation profiles.

## Discussion

The purpose of this study was to examine the implications of work motivation on retirement intentions and correlates of retirement intentions (e.g., vigor, stress, emotional exhaustion, and turnover intentions). While a number of factors can contribute to the decision to retire (Midtsundstad et al., 2017), work motivation is an important contributor to both retirement decisions, as well as other related factors also contributing to retirement such as work-related well-/and ill-being. Indeed, the results of the present study showed the importance of work motivation for senior workers’ retirement decisions in a representative sample of 500 Norwegian workers aged between 58 and 72.

This is the first person-centered study in the literature on retirement intentions. Another incremental contribution to the literature is the use of the full MWMS (Gagné et al., 2015), as previous research has omitted some of the regulations included in this scale or used older versions of the scale (Van den Broeck et al., 2013; Graves et al., 2015; Gillet et al., 2017, 2018; Fernet et al., 2020). In addition, the use of the state-of-the-art LPA and BCH approaches to analyses (Howard et al., 2016) in relation to profile detection and their outcomes is a strength. Finally, an important contribution of the current study is the use of a relatively large sample country-representative for all types of occupations strengthening the generalizability of the findings, as previous research has been based on one to three occupations or industry sectors (Howard et al., 2016; Gillet et al., 2017; Fernet et al., 2020).

The person-centered approach using LPA distinguished between four latent profiles of older workers, based on their work motivation regulations defined according to SDT as intrinsic, identified, introjected approach and avoidance, external social and material, and amotivation. The four profiles were: (1) The Low motivation profile characterized by average and below-average scores on most motivational regulations, but in particular very much below average on the identified regulation; (2) the Autonomous profile with above-average scores on autonomous types of regulations and below-average scores on controlled regulations and amotivation; (3) the High motivation profile with above-average levels of both autonomous and controlled types of regulations and below-average scores on amotivation; (4) the Amotivated profile with extremely high scores on amotivation, above-average to average controlled types of regulations, and below-average levels of autonomous regulations. The profiles and the correlations among motivational regulations observed in this relatively large country-representative sample of older workers supported the self-determined continuum structure proposed by SDT (Ryan and Deci, 2017). In addition, the current study replicated profiles found by others (Moran et al., 2012; Van den Broeck et al., 2013; Graves et al., 2015; Howard et al., 2016; Fernet et al., 2020), which are the autonomous, the highly motivated, and the amotivated profiles. The Low motivation profile with extremely low scores on identified regulation is a more novel finding, which may characterize older workers approaching retirement.

Based on SDT (Deci et al., 2017) and, in part, research using the full MWMS (Howard et al., 2016), we hypothesized that the most autonomous profiles (viz., autonomous and highly motivated) would be associated with higher levels of vigor and lower levels of job stress, exhaustion, turnover, and retirement intentions compared to the extreme profiles of low motivation and amotivation. The results confirmed our hypothesis. In addition, lower levels of retirement intentions were significantly associated with higher levels of vigor, and with lower levels of job stress, exhaustion, and turnover intentions, supporting our hypothesis and previous research (Nielsen Breidahl, 2011; Hofstetter and Cohen, 2014; De Wind et al., 2017; Stynen et al., 2017).

The Autonomous profile and the High motivation profile yielded the highest scores on vigor (viz., indication of job engagement), and the lowest scores on exhaustion, turnover, and retirement intentions. However, higher job stress was observed in the High motivation profile compared with the Autonomous profile. Both of these profiles scored equally high on the autonomous regulations (viz., intrinsic and identified), but compared to the Autonomous profile, the High motivation profile yielded significantly higher controlled types of regulations. That is, higher introjection approach (ES = 0.60), higher introjection avoidance (*ES* = 0.50), higher external social (ES = 0.61), and higher external material regulations (*ES* = 0.38). Hence, holding both higher autonomous and controlled types of work motivation are associated with higher job stress among older workers compared with the pure Autonomous profile with lower scores on controlled types of regulations. This finding is a novel finding regarding job stress and contributes with nuances to the picture presented in other studies (Van den Broeck et al., 2013; Howard et al., 2016) which have concluded that controlled types of regulations does not add to work-related outcomes or are unimportant when combined with autonomous types of motivation. Conversely, SDT and the findings in the present study indicate that controlled regulations characterized by ego-orientation and competition (viz., introjection approach), shame, guilt, and fear related to failure (viz., introjection avoidance), loss of acknowledgment from colleagues and managers (viz., external social), and fear of losing job and income (viz., external material), are associated with higher job stress even when autonomous types of motivation are present. To reduce job stress and increase positive job-related outcomes, managers and organizations may provide employees with job tasks stimulating meaningfulness, importance, interest, and cooperation, and tuning down the focus on ego-orientation, competition, and contingent rewards (Deci et al., 2017; Ryan and Deci, 2017). Consequently, in the long run, their autonomous work motivation is expected to be strengthened, increasing their vigor and reducing their job stress, exhaustion, and turnover intentions, and finally reducing their intentions to leave the workforce.

The Low motivation profile is a novel finding and becomes very interesting when comparing their intrinsic and identified regulations with the other profiles. Their very low identified work motivation, indicating loss of work identity, implies that these employees feel that their work has very low importance, personal value, meaning, and interest. Accordingly, their identified work motivation is very low (viz., large to very large ESs) compared with the Autonomous motivation profile, the High motivation profile, and the Amotivated profile. The findings indicate that their scores on most outcome variables are in between the highest-scoring Autonomous and High motivation profiles, respectively, and the Amotivated profile and their retirement intentions are higher than those found for profiles 2 and 3. Due to the very low levels of intrinsic motivation and identified regulation in the Low motivation profile, managers and organizations should offer and/or redesign these employees work in a way that maximizes their basic psychological need satisfaction, shown to be a key endeavor in order to stimulate increases in autonomous types of motivation, such as identified and intrinsic regulations, and decreases in controlled types of motivation, such as introjection and external regulation (Deci et al., 2017). According to SDT, managerial and organizational needs support would indeed satisfy employees’ basic psychological needs, and thereby increase employees’ autonomous work motivation and decrease their controlled work motivation, promoting an effective organization through the facilitation of employees’ well-being and performance (Baard et al., 2004; Paauwe, 2009; Deci et al., 2017; Stenius et al., 2017). The elements of needs support that managers and organizations can offer employees are (1) choices of work tasks and work redesign based on work interest and participation in decision making, supposed to maximize the support for the need for autonomy; (2) giving positive performance feedback in the process and offering training and education if relevant to maximize support of the need for competence; (3) relate to employees in a warm and caring way to maximize the support for the employees’ need for relatedness. A fuller description of the need-supporting behaviors supposed to increase autonomous motivation is considered by Ryan and Deci (2017) and by Halvari et al. (2021).

Compared to the Autonomous and High motivation profiles, the Amotivated profile reported significantly lower scores on vigor and significantly higher scores on job stress, exhaustion, turnover, and retirement intentions. This profile with extreme amotivation also performed more badly in relation to levels of vigor, job stress, and turnover than the Low motivation profile. Extreme scores on amotivation were combined with the highest scores observed on external regulations and also very low autonomous types of motivation, the latter similar to the Low motivation profile. This means that the same managerial and organizational endeavors suggested for the Low motivation profile to decrease their external and increasing their autonomous motivations also becomes relevant for this group of employees. However, their extreme scores on amotivation require a second look as these employees have no intention to put effort into their work. Amotivation results from at least two different sources. First, from a lack of perceived competence described by perceived behavior-outcome independence (e.g., “My job behavior will not yield desired work outcomes”) and/or a perceived work behavior incompetence (e.g., “I cannot perform adequately”). A second source is not so much related to competence, but more autonomy-related involving employees who perceive a lack of value, meaning, and interest in their work. They are indifferent toward their work activities and their outcomes and do not care to act (Ryan and Deci, 2017). Hence, in the present study, amotivation seems to have more to do with the second source described above than the first source. This is because amotivation is combined with very low levels of autonomous motivation, just described with low levels of value, meaning, and interest. Conversely, amotivation is also combined with the highest levels of external social and material regulations, and consequently, being non-intentional or amotivated in their work behavior is most likely controlled. This is also emphasized by the Amotivated profile demonstrating the highest levels of job stress, exhaustion, turnover-, and retirement intentions.

Using the alternative measure of retirement “planned length of working life in years,” estimated differences between profiles indicated that the Autonomous and High motivation profiles planned to work for about 1.6–3.4 years longer (adjusted for standard errors) than the Low motivation profile and the Amotivated profile. Hence, it is interesting and important to register that a 1-year increase in professional activity among the population 60 years and above is estimated to increase the income for the Norwegian State by about 94 billion NOK, equivalent to 0.01% of the Gross Domestic Product.

## Limitations and Directions for Future Research

The current study has certain limitations that one should bear in mind when interpreting the results. Acknowledging the multitude of factors involved in senior employees’ decisions to retire or continue working, future studies should include variables not examined in the present study, such as factors related to family, leisure, health, planning of retirement, and close relationships (Noone et al., 2013; Midtsundstad et al., 2017). The data is cross-sectional in nature and can, thus, not imply any causality among the study variables (Cook et al., 2002). Furthermore, longitudinal data could strengthen the findings regarding changes over a longer period of time (e.g., using growth curves; Mäkikangas et al., 2010). As this is the first known study to explore motivational profiles in a sample of older workers and their relations to the selected outcome variables, it is, of course, necessary for the study to be replicated with other samples. These future studies should include antecedents of motivational profiles, which may help to understand mechanisms that would increase the likelihood of being more autonomously motivated at work, and thus, stimulate older workers to stay for longer in the workforce.

## Data Availability Statement

The raw data supporting the conclusions of this article will be made available by the authors, without undue reservation.

## Ethics Statement

Ethical review and approval was not required for this study on human participants in accordance with the local legislation and institutional requirements. The data was collected by NORSTAT Market Research AS, approved by the ethics committee of Norway. The patients/participants provided their written informed consent to participate in this study.

## Author Contributions

All authors listed have made a substantial, direct, and intellectual contribution to the work, and approved it for publication.

## Conflict of Interest

The authors declare that the research was conducted in the absence of any commercial or financial relationships that could be construed as a potential conflict of interest.

## Publisher’s Note

All claims expressed in this article are solely those of the authors and do not necessarily represent those of their affiliated organizations, or those of the publisher, the editors and the reviewers. Any product that may be evaluated in this article, or claim that may be made by its manufacturer, is not guaranteed or endorsed by the publisher.
